# Supplementary material for: Exploring, measuring and enhancing the coproduction of health and well-being at the national, regional and local levels through comparative case studies in Sweden and England: the ‘Samskapa’ research programme protocol
Source: BMJ Open. 2019 Jul 26;9(7):e029723. doi: 10.1136/bmjopen-2019-029723 (PMC6661680; doi:10.1136/bmjopen-2019-029723)
Supplement: Author's manuscript [file bmjopen-2019-029723.draft_revisions.pdf]

BMJ Open

BMJ Open is committed to open peer review. As part of this commitment we make the peer review history of every article we publish publicly available.

When an article is published we post the peer reviewers' comments and the authors' responses online. We also post the versions of the paper that were used during peer review. These are the versions that the peer review comments apply to.

The versions of the paper that follow are the versions that were submitted during the peer review process. They are not the versions of record or the final published versions. They should not be cited or distributed as the published version of this manuscript.

BMJ Open is an open access journal and the full, final, typeset and author-corrected version of record of the manuscript is available on our site with no access controls, subscription charges or pay-per-view fees (http://bmjopen.bmj.com).

If you have any questions on BMJ Open's open peer review process please email info.bmjopen@bmj.com

BMJ Open

Exploring, measuring and enhancing the co-production of health and wellbeing at the national, regional and local levels through comparative case studies in Sweden and England: the Samskapa research programme

Journal:	*BMJ Open*
Manuscript ID	bmjopen-2019-029723
Article Type:	Protocol
Date Submitted by the Author:	07-Feb-2019
Complete List of Authors:	Kjellstrom, Sofia; Jonkoping University, Jonkoping Academy<br>Areskoug-Josefsson, Kristina; Jonkoping University, Jonkoping Academy<br>Gäre, Boel Andersson; Jonkoping University, Jonkoping Academy<br>Andersson, Ann-Christine; Jonkoping University, Jonkoping Academy<br>Ockander, Marlene; Jonkoping University, Jonkoping Academy<br>Kall, Jacob; Djursdala<br>McGrath, Jane; We Coproduce<br>Donetto, Sara; King's College London, Florence Nightingale Faculty of Nursing, Midwifery & Palliative Care<br>Robert, Glenn; King's College London, Florence Nightingale Faculty of Nursing, Midwifery & Palliative Care; Jonkoping University, Jonkoping Academy
Keywords:	Health policy < HEALTH SERVICES ADMINISTRATION & MANAGEMENT, Protocols & guidelines < HEALTH SERVICES ADMINISTRATION & MANAGEMENT, co-design, co-production, social care

SCHOLARONE™
Manuscripts

Exploring, measuring and enhancing the co-production of health and wellbeing at the national, regional and local levels through comparative case studies in Sweden and England: the 'Samskapa' research programme

Sofia Kjellström, The Jönköping Academy for Improvement of Health and Welfare, Jönköping University, Gjuterigatan 5, Box 1026, 551 11 Jönköping, Sweden, sofia.kjellstrom@ju.se +46 36-10 1313

Kristina Areskoug Josefsson, The Jönköping Academy for Improvement of Health and Welfare, Jönköping University, Jönköping, Sweden

Boel Andersson Gäre, The Jönköping Academy for Improvement of Health and Welfare, Jönköping University, Jönköping, Sweden

Ann-Christine Andersson, The Jönköping Academy for Improvement of Health and Welfare, Jönköping University, Jönköping, Sweden

Marlene Ockander, The Jönköping Academy for Improvement of Health and Welfare, Jönköping University, Jönköping, Sweden

Jacob Käll, Djursdala, Sweden

Jane McGrath, we coproduce, London, United Kingdom

Sara Donetto, Florence Nightingale Faculty of Nursing, Midwifery & Palliative Care, King's College London, London, United Kingdom

Glenn Robert, Florence Nightingale Faculty of Nursing, Midwifery & Palliative Care, King's College London, London, United Kingdom ORCID 0000-0001-8781-6675

Word count (excluding title page, abstract, references, figures and tables): 4,093 words

For peer review only - http://bmjopen.bmj.com/site/about/guidelines.xhtml

Exploring, measuring and enhancing the co-production of health and wellbeing at the national, regional and local levels through comparative case studies in Sweden and England: the Samskapa research programme

Abstract

Introduction: co-creation, co-production and co-design are advocated as effective ways of involving citizens in the design, management, provision and evaluation of health and social care services. Although numerous case studies describe the nature and level of co-production in individual projects, there remain significant gaps in the evidence base. Our overall aim is to explore, enhance and measure the value of co-production for improving the health and wellbeing of citizens. We will focus on three fundamental issues: (a) measures of co-production processes and their outcomes, (b) mechanisms that enable inclusivity and reciprocity, and (c) management systems and styles.

Methods and analysis: nine confirmed co-production projects will form the core of an interactive research programme ('Samskapa**)** during a six-year period (2019-24). Six of these will take place in Sweden and three will be undertaken in England to enable knowledge exchange and cross-cultural comparison. The programme has a longitudinal case study design using both qualitative and quantitative methods. Cross-case analysis and a sensemaking process will generate relevant lessons both for those participating in the projects and researchers. Based on the findings we will develop explanatory models and other outputs to increase the sustained value (and values) of future co-production initiatives in these sectors.

Ethics and dissemination: all necessary ethical approvals will be obtained from the regional Ethical Board in Sweden and from relevant authorities in England. All data and personal data will be handled in accordance with General Data Protection Regulations. Given the interactive nature of the research programme, knowledge dissemination to participants and other stakeholders will be ongoing throughout the six years. An additional dissemination mechanism to practitioners and wider interest groups will be through external workshops in collaboration with participating case studies and citizens both during and at the end of the programme.

Keywords: co-production, co-design, healthcare, social care, case study

For peer review only

Strengths and limitations of this study

- moving beyond the study of individual co-production projects and taking a longitudinal, multi-level, cross-case approach to explore the complexities of enabling new forms of relationships to improve health and wellbeing
- a research group from different disciplines and professional backgrounds including interdisciplinary social science, nursing, medical anthropology, medicine, rehabilitation, and sociology
- a close partnership with practitioners and patients/citizens through an interactive research approach will help co-produce and co-evaluate the programme itself
- a potential limitation is the socio-cultural and linguistic differences between the two countries in which the fieldwork will take place

For peer review only - http://bmjopen.bmj.com/site/about/guidelines.xhtml

Introduction

As in many other countries the Swedish health and social care system is struggling to balance contemporary challenges of increasing demands and rising costs resulting from demographic changes with the opportunities afforded by technological and scientific advances. As Palmer et al suggest, within health system re-design and quality improvement internationally there is currently a 'preoccupation with methods for citizen engagement, public participation and involvement of people with lived experience … Participation has become a distinct cultural and political movement characterised by user involvement in health and social care'.[1] The forms of such involvement span shared decision-making, person-centred care and management at the individual patient level through quality improvement, research and evaluation at the service and organisational levels, and to policy-making at the system level.

Principles underpinning the concept of co-production - such as mutuality and reciprocity - offer the possibility of fundamentally challenging and changing predominant ways of thinking by moving from focusing solely on the delivery of healthcare and social care and towards co-creating health and wellbeing. This is evidenced in successful, long-standing co-production projects - for example, the co-creation of a self-hemodialysis service, see table 1) - undertaken over several years in the Region Jönköping County, by the development and adaptation internationally of approaches such as Experience-based Co-design (EBCD)[2], and through the work of third-sector organizations like 'We Coproduce' in England[3].

Table 1 A co-production initiative from Region Jönköping County

**Origin:**<br>In 2005 a young engineer asked his nurse to teach him to set up and run the hemodialysis he needed. Later other patients wanted to join. A learning system for patients to manage their self-dialysis, a new unit and an innovative co-productive way of working was created.<br>**Aim:**<br>To enhance quality of life and clinical outcomes for patients by allowing them to control their hemodialysis treatment.<br>**Basic principles:**<br>• to give patients control of their lives and self-hemodialysis through self-management.<br>• create hospital units where this is done and support the quality and safety of the procedure.<br>**Timeline:**<br>2005: started with one person<br>2010: building a unit, where patients were involved in design of the "learning ladder" and in the purchasing of new dialysis machines.<br>2010 onwards: spread across hospitals in the region and later nationally and internationally. Widespread interest from national and international groups (Institute for Healthcare Improvement, American Association of Physicans)

There has recently been renewed academic interest in - and advocacy for - adoption of 'co-production' as a means of co-creating value across the public sector[4,5]. In the healthcare context,

For peer review only - http://bmjopen.bmj.com/site/about/guidelines.xhtml

co-production is promoted as harnessing the knowledge of patients, carers and staff to make changes about which they care most. It is claimed that 'bringing people together to re-design and improve services as co-producers is re-creating the ways in which public governance, policy and health services are enacted and function'.[1]

Origins and evolution of co-production

Originating in the early 1970s, the term co-production refers to how citizens themselves play an important role in determining the form, delivery and value of public goods and services [6,7]; academic studies of the time were a response to what was seen as a lack of recognition of the role of service users in determining the relative effectiveness of service delivery in different local contexts[8,9]. The creation of time banks, a system reliant on the participation of volunteers who are also service users[10,11], popularised how collaborative interventions that involve people with long-term psychosocial needs could contribute to improved community links[12].

Academic interest in co-production in different sectors has waxed and waned over the subsequent 50 years but the concept is currently attracting greater interest in public management practices generally[13], including in the healthcare sector specifically. Much of this interest is based on claims of better outcomes and/or efficiency arguments despite empirical evidence remaining limited[14]. With contemporary practices of public sector service delivery highlighting - and espousing the benefits of - efforts to enable co-production has come renewed critical interest from a range of academic disciplines. As summarised by Palmer et al[1], commentators have argued that whilst such interest may reflect a genuine desire to engage citizens in democratic processes, governance and decision-making[15], it can also be variously perceived as a means to harness citizen efforts and resources as a replacement for reduced public funding (the 'dark side' of co-production)[16], as a representation of a loss of public value and trust in public sector services and/or a drive to reinvigorate voluntary participation and strengthen community cohesion in response to increasing societal fragmentation.[17] In the healthcare sector specifically, it has been argued that increasing interest in efforts to enable co-production remain uncritical, lacking acknowledgement of the ethical complexities embedded in welfare [and service] relationships.[17] Others have warned against the risk of the term itself losing meaning as it enters mainstream management discourse and practice, thereby losing association with its radical roots.[18] For the purposes of this research programme we are following Osborne et als definition of co-production as 'the voluntary or involuntary involvement of public service users in any of the design, management, delivery and/or evaluation of public services'.[19]

Co-production in the health and social care sector

Notwithstanding definitional issues, Palmer et al argue that we are witnessing 'a political and socio-cultural mindset shift from ''experts know and decide everything'' to ''we need to decide things together''.[1] In the context of health services, people are increasingly characterised as designers, learners, and actors who can take responsibility for their own health and shape the outcomes that they desire from organisations.[2,4,5] An excerpt from a recent manifesto for a third (and moral) era in medicine and health care is typical of this shift: 'Co-production', 'co-design' and person-centred care are among the new watchwords, and professionals and those who train them, should master those ideas and embrace the transfer of control over people's lives to the people."[20] Whilst such contemporary interest in co-production from leaders of quality improvement and Improvement Science in the health and social care context is a relatively recent development[21,22], advocates of the need to study and learn from co-production across the public sector have been doing so for some time.[11,23] A Swedish Patient Act supports these trends[24] but there is also a concern that

For peer review only - http://bmjopen.bmj.com/site/about/guidelines.xhtml

'co-production' may become a much used but ultimately meaningless term that everyone says that they can and want to do but without understanding its origins or how to practice and evaluate it.

Knowledge gaps

We highlight below 3 significant and interrelated knowledge gaps pertaining to co-production in the health and social care sectors which our research programme seeks to address:

- outcomes (measurement),
- power, power relations and representation (mechanisms)
- leadership (management)

Firstly with regard to measurement, Voorberg et al conducted a systematic review of the literature (1987–2013) relating to co-creation/co-production with citizens in public innovation across all sectors, concluding that most studies focus on the identification of influential factors, while hardly any attention was paid to outcomes.[13] Similar conclusions have been drawn by others.[14] Clarke et al systematically reviewed outcomes associates with developing and implementing co-produced interventions in acute healthcare settings and found a lack of rigorous effectiveness and cost-effectiveness studies at both the service and system levels.[18] Greenhalgh et al report a narrative review of different models of co-creation relevant to community-based health services; they identify key success principles, such as system thinking, processes of co-creation and leadership styles, but note that 'impact is by no means guaranteed'.[25]

Secondly, academic reviews of co-production highlight - with some exceptions[26] - little critical engagement with issues of power, power relations, and representation and whether typical patterns of participation serves to make services more or less inclusive (by simply reinforcing existing social inequalities).[4,13] In a recent commentary, Batalden et al - while arguing that where healthcare activities are co-produced, services, providers and service users become far more effective agents of change - note that current systems can both support and constrain partnerships between patients and professionals (and that historically this kind of partnership has been unequal).[22]

Thirdly, management or leadership of co-production activities are characterized by the involvement of many stakeholders sharing different perspectives on the same issue and yet, despite being described as a founding principle, studies of leadership in co-production initiatives are sparse[27]; this is a neglected area[28,29]. A recent review of the leadership literature in relation to co-production suggests four main challenges[27]:

- setting priorities of co-production and clarifying goals;
- guaranteeing greater inclusion of vulnerable and disadvantaged populations;
- fostering communication and public accountability; and
- encouraging and supporting innovative practices and cultural changes that move away from traditional challenges of risk aversion.

This suggests that the leadership of co-production initiatives over time involves several practices, emerges as a complex and collective activity (rather than relying on individual leaders), and likely requires a facilitative leadership system and style.[27]

In summary, despite a recent resurgence of interest in the potential of co-creation and co-production as means not only of maintaining but also improving health and wellbeing in Sweden and England, there remain significant questions regarding how to measure the impacts of such

For peer review only - http://bmjopen.bmj.com/site/about/guidelines.xhtml

approaches, the mechanisms by which they achieve those impacts and how they can and should be led.

Aim

The overall aim of the Samskapa research programme is to explore, enhance and measure the value of co-production for improving the health and wellbeing of citizens. Our four research objectives are:

1. to develop, test and establish robust measures of co-production processes and of the outcomes of co-production with participants and wider constituencies at different system levels (measurements)
2. to study the social processes and organisational forms that enable inclusive and reciprocal co-production across and beyond the health and social care sectors (mechanisms)
3. to explore the features of effective systems and styles of leadership that are necessary to enable co-production (management)
4. to develop explanatory models and other outputs based on a synthesis of existing evidence, analysis of our case studies and our findings in relation to 1-3 above in order to help enhance future co-production initiatives (model).

Methods and analysis

This is a six-year, interactive research programme[30] (see figure 1) beginning in 2019 that will provide a platform for mixed method evaluations of at least nine case studies of co-production in the health and social care sectors (see table 2 for details of the case studies). The interactive research approach will place emphasis upon joint learning between the participants and the researchers throughout the entire research process, from the definition of the issues to the analysis and dissemination of findings. Cross-case analysis and a sensemaking process with participants and researchers from the case studies will lead to practical lessons and outputs to assist practitioners, as well as helping address the key research gaps in the current evidence base as identified above: measurement, mechanisms and management.

As well as responding to recent calls for a 'more multidisciplinary framework, using social-psychological, organizational and institutional theories' to form the basis for future co-production research[4], the programme has a longitudinal design and will use both qualitative (semi-structured interviews, non-participant observation, focus groups) and quantitative methods (for example, patient reported outcome measures and patient reported experience measures within feasibility studies of co-designed interventions). Through longitudinal case studies we will seek to establish - and enhance - the impact of co-production, as well as exploring the complexities of delivering and improving health and social care through new forms of relationships and partnerships.

[INSERT] Figure 1: Interactive research

Study setting

For peer review only - http://bmjopen.bmj.com/site/about/guidelines.xhtml

Fieldwork will be conducted in the context of health and social care provision in Sweden and England. The case studies in Sweden will be undertaken in Region Jönköping County, Kalmas, Stockholm and Gothenburg; the English case studies are in London.

Project management

Four research-focused work packages will help to deliver on each of our study objectives. Three further management-related work packages will focus on, firstly, coordinating the work of the program as a whole; secondly, building doctoral and post-doctoral research capacity and capability for studying co-production in Sweden; and thirdly the continuous dissemination of our findings.

A narrative literature review

We will begin the programme by conducting a narrative literature review to explore current knowledge about co-producing health and wellbeing in developed countries, establish best practice within the health and social care sectors, and identify research priorities for the Samskapa programme. Specific objectives within the review will relate to each of our four objectives, as outlined below.

The case studies

Nine case studies of co-production have initially been proposed as the basis for the programme (table 2). This initial set of case studies is an opportunistic sample and six case studies are the subject of planned or ongoing doctoral studies in Sweden or England. There is scope during the six years of the programme to purposively add further case studies as they emerge and in order to help meet the four research objectives. The co-production efforts and processes in each case will be explored by documentary analysis as well as qualitative) and quantitative methods as relevant to the objectives of each case study.

Table 2 Initial nine case studies (in Sweden unless stated)

The Esther network	The Esther network - coordinated by Region Jönköping County - recently won the ICT-Enabled Social Innovation (IESI Award) from the EU Science Hub for best initiative supporting active and healthy ageing. The network aims to improve patient flow and coordination of care. The award was for its positive contribution to society as well as its disruptive ICT enabled social innovation potential and high level of service integration. From its origins in 1997 the Esther model has subsequently been adopted and implemented in England, Scotland and Singapore. Our research will focus on learning how local co-production approaches can be spread and implemented internationally whilst addressing key issues of fidelity and adaptation.
Patient compact	At the local level, an ongoing strategic innovation program in Region Jönköping County (the 'Together' programme) started in 2012 and has developed and expanded over time. The purpose of the programme is to transform health care delivery closer to citizens, from hospitals to primary care, from primary care to home care and with a focus on health promotion efforts together with other community actors and citizens themselves. The programme is divided into several subprojects, of which the development and implementation of a 'Patient Compact' is one. In combination with a national initiative to introduce a 'Patient Compact' throughout Sweden, different interactions at different levels will be studied, aiming to enhance emerging understandings of co-production as they evolve over time within the

For peer review only - http://bmjopen.bmj.com/site/about/guidelines.xhtml

	Together program and establish measures relating to co-production and its potential impact on health and wellbeing.
We Coproduce (UK)	The origins of We Coproduce as a social enterprise in 2013 are rooted in the recognition, at a mental health hospital in London, that service user involvement was not working, with subsequent development of an independent social enterprise. We Coproduce then also began to work with other community organisations and providers across London to help them embed co-production in their service design and delivery. Ongoing projects include: coproducing a community owned and run radio station; coproducing with frontline mental healthcare staff to support them to make their own films about trauma-based approaches; and coproducing with a local council to embed micro businesses in partnership with bigger local businesses to challenge isolation. Our research with We Coproduce will focus on exploring challenges and opportunities of service-user led co-production projects in local communities.
Djursdala community project	This case study is funded by an European Union initiative to support rural development projects initiated at the local level in order to revitalize rural areas and create employment opportunities. It seeks to identify needs and initiate the development and use of digital solutions that promote the health and wellbeing of a population of approximately 400 citizens. Staff at Jönköping Academy are coordinating research into this initiative which is led by the local community. A pre-study was completed in August 2018 and a tool for the local community to follow their own data has been coproduced. We will explore, firstly, how can user-driven digital development enable co-created and co-produced services that lead to value for a rural area, and secondly, do digital solutions contribute to sustainable development and, if so, in what way?
Chemotherapy-induced peripheral neuropathy (UK)	This study aims to co-design and test an intervention to reduce falls and injuries and improve functional status and quality of life among individuals with chemotherapy-induced peripheral neuropathy (CIPN). Some cancer drugs cause damage to nerves, a condition called CIPN. The most common symptoms, felt mainly on hands and feet, are numbness, tingling, pain, muscle weakness, and/or sensitivity to cold. People with CIPN can have functional difficulties in carrying out tasks involving their hands and feet. It is important to prepare patients about the possibility of developing CIPN to help them recognise and report symptoms early so healthcare professionals (HCPs) can support them. We will study how co-designed interventions can be developed and put in place early to prevent subsequent CIPN-related falls and injuries, reduce costs to healthcare systems and lessen the burden on HCPs and services.
Learning café: cardiac care	A Learning Café project is exploring whether, how and why the co-production of healthcare services, particularly for individuals (and their families) with cardiac care needs, can contribute to high quality care. A specific focus of the research is to explore what role motivation plays for patients, family members and healthcare professionals when co-producing healthcare. Clinical measures - as well as patient-reported outcome measures and patient-reported experience measures - will be co-designed with patients and families and professionals. In addition a co-designed conceptual model of the Learning Café which can be adapted to other groups of patients with chronic disease will be developed. For national

For peer review only - http://bmjopen.bmj.com/site/about/guidelines.xhtml

	dissemination the findings from this case study will also be linked to the national quality registry system in Sweden.
Disabled children & adolescents	This study is investigating whether and how children and adolescents with disabilities in supported living accommodation can work with staff to coproduce individual support and goal fulfilment. The study is taking place in a not-for profit organization in Solberga By, near Stockholm. A particular focus in our research will be to explore how the co-production process affects staff behavior and whether and how the 'International Classification of Functioning, Disability and Health' can be used as a framework for measures for establishing and following personal outcomes for children.
Therapeutic engagement on an acute psychiatric ward (UK)	This project in London is seeking to empower a service-user group to take a lead role, and in partnership with NHS staff, co-design and implement an intervention to improve nurse-patient therapeutic engagement on acute mental health wards. The project is being assessed in terms of: improvements in the amount, type and quality of nurse-patient engagement; improved service user/service provider relations; and the fostering of a culture of collaborative working/research practices within a psychiatric ward.
Learning health system for severe mental illness	In the department for psychosis at Sahlgrenska University Hospital in Gothenburg a Learning Health System has begun to be developed and tested along with patients, case-managers and the management team. Patients and families are active participants in considering system design, user-experience-design, choice of outcome measures and development of care processes. This project is seeking to enable learning throughout the whole "system" and continuous improvement. We will focus on the role of patients in, firstly, the development of data-visualization-design and how this impacts upon learning both for the patient and their case manager, and secondly in evaluating outcome measures useful for the patient and their case-manager in ongoing treatment.

Cross-case analyses

Based on the multi-level and longitudinal case studies in different health and social care settings in Sweden and England we will conduct a meta-synthesis.[32,33,34,35] We will use theoretical perspectives - identified and selected through a narrative literature review (see above) - and systems thinking to make sense of the context and cases.[36,37] In the meta-synthesis we will explore patterns in both the qualitative and quantitative data from across the different case studies and examine and compare differences and similarities across these. The intention is to examine relevant themes, similarities, and differences between the cases to inform each of the four research objectives (figure 2).

[INSERT] [Figure 2: Cross-case analyses]

For peer review only - http://bmjopen.bmj.com/site/about/guidelines.xhtml

Measurement

A narrative review will initially be conducted to explore how measures relating to participating in co-production processes and the outcomes of those processes have been developed, by whom and how they have been tested and applied (for example, Durose et al[38]). We will explore the usefulness of existing measures in the case studies by observing their use (if any) in practice and facilitating interactive workshops with case study leaders on this topic (particularly on how measures and co-evaluation of processes and outcomes are integrated in the cases). We will then co-design with stakeholders and users of the relevant services new generic measures of co-production (through an interactive process partly informed by the narrative review findings) and then test them over time in the case studies to assess their pragmatic usefulness and generalizability.

How - and to what extent - client/patient involvement is present and acted upon in the design and evaluation of outcome measures, as well as the sustainability and understanding of the value of the outcomes, will also be explored in the case studies. We will consider the gender and sociodemographic differences (for example, age, gender) amongst those who actively choose to participate in the co-production case studies as this may influence both the chosen measurements and the outcomes.[39] The programme will provide an increased understanding of how to develop and use measures within – as well as to evaluate the outcomes of - co-production projects by illuminating how specific processes in co-production relate to measured outcomes (see 'mechanisms' below).[39]

Mechanisms

We will study the concepts and practices of co-production to identify mechanisms (for example, social processes and organisational forms) that contribute to or hinder the development of values and actions that enable inclusive and reciprocal co-production. Our narrative review will firstly identify previous studies (for example, Palmer et al[1]) which have sought to explore such mechanisms as well as what is already known about key issues such as power and levels of representativeness, and any interventions or modifications that have attempted to resolve these. Secondly, we will draw on the narrative review findings and the emerging local conceptualisations and practices in the nine case studies to design complementary or alternative ways of working; we will subsequently test these in one or more of the case studies to see whether they enhance the co-production efforts.

Management/leadership

Our narrative review will establish what is known about different individual and collective leadership and management beliefs and practices that enable the co-production of health and wellbeing. Leadership will be framed as complex, interactive and dynamic, and analysed in a way that contributes to generative learning and theoretical transferability. [37,40] Studying leadership needs to shift from a linear focus on leaders and followers and towards a focus on processes in which the whole collective engages. The longitudinal design will enable us to address questions like: i) how do leadership beliefs and practices change and develop over time? And ii) how do people in different positions of power participate in various leadership practices?[41] We will explore leadership through a range of methods including semi-structured interviews, surveys, and non-participant observation of meetings and events. Workshops will also be facilitated with researchers and key actors to elicit their views on how leadership has been enacted in each of the cases. The results will be summarized in a dynamic system assessment tool that seeks to aid understanding of how to analyse and improve co-production leadership.

For peer review only - http://bmjopen.bmj.com/site/about/guidelines.xhtml

Models

This work will develop explanatory models for successful co-production through analysis of all cases and synthesis of our findings in relation to measurement, mechanisms and management. Further outputs will include heuristic tools for reflecting upon the maturity of co-production efforts. The narrative review will establish which and how explanatory models have been developed, tested and applied with the aim of enhancing co-production processes and outcomes. As a part of our interactive approach we will convene and facilitate multiple stakeholders meetings (with leaders, participants and researchers of the 9 case study projects) in the format of Joint Interpretive Forums (JIFs) - a form of group discussion which aims to foster 'perspective taking' and joint decision making - to enable the collaborative interpretation of both our review and empirical research findings and the development of actionable recommendations for policy and practice.[42] The first JIF will be convened at the end of year 3; a JIF will then be held at 6-month intervals for the remaining duration of the programme (7 JIFs in total). The initial and final JIF will be open to all participants whilst the others will focus on specific aspects of the emerging model(s) and invitees will be selected as appropriate. Informed by the findings from above all the JIFs will be held in Sweden. Importantly, all PhD students who are studying one of the case studies will be integral members of this ongoing sensemaking process giving them further personal development opportunities.

Patient and Public Involvement

Jönköping Academy for Improvement of Health & Welfare (JA) is the host of the research programme and has an established tradition of interactive research where knowledge is created in the interaction between theory and practice. 'Practice' has traditionally been represented by professionals in this model but in this programme we will use and expand this to include citizens and patients as full partners (see Figure 1). Processes to enhance partnership working through ongoing, joint design of the specific research materials and methods to be applied in each of the case studies will therefore be both a result and a phenomenon for study in the programme. As part of our ongoing interactive approach, members of the research team have identified several 'charters for co-production' (tools to aid reflective practice within co-production projects) from the UK and the US and had these translated into Swedish. These will form the basis for reflective dialogues which will test the appropriateness of applying these various materials in the Swedish context.

Through conversations with public and third-sector organizations already engaged in co-production in health and social care, the Region Jönköping County in 2016 decided to financially support development of an International Centre for Co-production hosted by JA as a sister Centre to a similar Centre at the Dartmouth Institute, New Hampshire in the United States. In 2017 Jönköping University made a strategic decision to support the research capacity in the Centre at JA through investing in senior research positions in co-production and a project leader. In addition, trade unions and small and medium size enterprises have been involved in ongoing discussions. Processes to integrate user and public representatives are in progress and they can contribute in several ways; in the co-design of service innovations but also in interactive research processes to assure the relevance of questions and the validity of results. The Centre provides infrastructure that supports the involvement of patients and citizens in research processes as well as in practice. From the UK, a community-based organisation 'We Coproduce' will form one of our case studies and leaders of this organisation will also engage with the doctoral and post-doctoral students in a regular series of practice-based workshops. The leaders of the Djursdala and 'We Coproduce' case studies have contributed to and are co-authors of this protocol.

For peer review only - http://bmjopen.bmj.com/site/about/guidelines.xhtml

Partnership working is an inherent feature of each of the nine case studies of co-production which we will initially be studying. At the programme level, a Partnership Learning Group will consist of all the current authors, participating post-doctoral researchers and doctoral students, as well as practitioners and citizens from the six Swedish case studies. This group will meet throughout the six-year programme. Through dialogue, cross-case study sharing of knowledge and development of further research questions and thoughts of interest, the members will help co-design, co-produce, and co-evaluate the program as a whole; these interactions will be supported by specific seminars. The Group will design dissemination workshops within each of their case studies to enhance cross-case study knowledge creation and networking.

Ethics and dissemination:

Ethics

Ethical approval will be obtained from the regional Ethical Board in Sweden for all of the case studies where this is applicable and from the relevant authorities in England for the case studies there. All data and personal data will be handled in accordance with the new European General Data Protection Regulation (GDPR) (EU) 2016/679). Given the need to respect the integrity, autonomy, and privacy of the participants, it is important to acknowledge that ethical considerations and principles may exert an influence on the research design. Informed consent will be accomplished by mutual communication where the researcher provides accurate information and listens to the individual participants in order to make sure that they comprehend and make voluntary choices to participate, not only at initial recruitment but also throughout participation in the project. This is particularly important for vulnerable groups,[43] some of which will be participating in this programme. Whilst the interactive research design (i.e. including patients, staff and stakeholders in the whole research process) can be challenging to Ethical Boards - posing difficulties in detailing the nature and timing of the research to be undertaken beforehand - the research team have extensive experience of successfully navigating these ethical processes in both Sweden and England.

Dissemination

Our intention is to engage strategically with five audiences with whom we will deepen and sustain existing - and create new - relationships to help inform our ongoing research and to provide opportunities to create positive change in health and social care services:

- leaders in the international, national and regional planning of health and social care services
- educators developing the next generation of health and social care professionals
- professional bodies and trade unions
- citizens as they access, use and shape services
- research funders

We will adopt a structured approach to mapping key organisations, networks and opinion leaders at international, national and regional levels; this will be part of our work both in terms of developing a communications strategy in year one of the programme and to help us identify diverse and influential members of our Advisory Board. As part of this approach we will engage with national and regional leaders of health and social care services through contact with the 'Swedish Association of Local Authorities and Regions' (SALAR). The Department for Health & Social Care at SALAR has responsibility, for example, for supporting the development of elderly care, social care, public health, disability, quality and safety, and equity. SALAR also coordinates several national networks of

For peer review only - http://bmjopen.bmj.com/site/about/guidelines.xhtml

leaders from across all Swedish regions and counties providing opportunities to share our findings and outputs to contribute to policy discussions and practice.

A further prime opportunity for dissemination is through involving educators and students - often mid-career professionals - involved with a Master programme in 'Leadership for Improvement of Health and Welfare' developed and run at the Jönköping Academy, Jönköping University; this will enable us to engage with students involved in leading improvement of care throughout Sweden. We also have connections with national bodies with an interest in integrating knowledge on co-production into different levels of education. A process of interaction with professional bodies and trade unions started in March 2018 and will enable further collaboration during the research programme. Citizens are both key participants in - and an important audience for - the programme and will be invited to participate and integrated throughout our work; we will also approach Swedish patient organizations. Whilst there is no national umbrella patient organization, there are many disease/condition specific patient advocacy groups which offer opportunities for testing and scaling up co-production initiatives. Finally, contact has also been initiated by the funder with those leading parallel research programs on co-production taking place in Sweden raising the potential for increasing adoption of the findings within and across programmes.

In combination with the interactive research approach outlined above, the proposed involvement of researchers, practitioners and citizens will enable fruitful ways of dissemination and impact throughout the six year Samskapa programme, enhancing the likelihood that co-production can be a catalyst for new forms of relationships to deliver and improve health and wellbeing.

References

1 Palmer V, Weavell W, Callander R, et al. The participatory Zeitgeist - identification of mechanisms of change and the development of an explanatory theoretical model for a new era of healthcare quality improvement. *Medical Humanities* Published Online First: 28 June 2018. doi: 10.1136/medhum-2017-011398

2 Robert G, Cornwell J, Locock L, et al. Patients and staff as co-designers of health care services. *British Medical Journal* 2015; 350:g7714

3 we coproduce. https://www.wecoproduce.com/, accessed 23/01/19

4 Brandsen T, Steen T, Verschuere B (Eds). Co-production and co-creation. Engaging citizens in public services. NY: Routledge 2018

5 Pestoff V, Brandsen T, Verschuere B (Eds). New Public Governance, the Third Sector and Co-Production. NY, Routledge 2012

6 Ostrom E, Parks RB, Whitaker GB, et al. The public service production process: a framework for analysing police services. *Policy Studies Journal* 1978; 7: 381-9

7 Alford J. The Multiple Facets of Co-Production: Building on the work of Elinor Ostrom. *Public Management Review* 2014; 16(3):299-316

8 Realpe A, Wallace LM. What is co-production? London: The Health Foundation 2016

9 Alford J. Engaging public sector clients, from service delivery to co-production. NY: Palgrave 2009

10 Boyle D, Clark S, Burns S. Hidden work: co-production by people outside paid employment London: Joseph Rowntree Foundation 2006

11 Needham C, Carr S. Co-production: an emerging evidence base for adult social care transformation. London: Social Care Institute for Excellence 2009

For peer review only - http://bmjopen.bmj.com/site/about/guidelines.xhtml

For peer review only

12 Alford J, Freijer L. Public management and co-production. In: Brandsen T, Steen T, Verschuere B (Eds). Co-production and co-creation. Engaging citizens in public services. NY: Routledge 2018

13 Voorberg WH, Bekkers VJJM, Tummers LG. A Systematic Review of Co-Creation and Co-Production: Embarking on the social innovation journey. *Public Management Review* 2015; 17:9: 1333-1357

14 Loeffler E, Bovaird T. User and Community Co-Production of Public Services: What Does the Evidence Tell Us? *International Journal of Public Administration* 2016; 39(13): 1006-1019

15 Brandsen T, Honingh M. Distinguishing Different Types of Co-production: A Conceptual Analysis Based on the Classical Definitions. *Public Administration Review* 2016; 76(3):427-35

16 Fotaki M. Co-production under the Financial Crisis and Austerity: a means of democratizing Public Services or a race to the bottom? *Journal of Management Inquiry* 2015; 24(4):433-8

17 Cribb A, Gewirtz S. New welfare ethics and the remaking of moral identities in an era of user involvement. *Globalisation Societies and Education* 2012; 10(4):507-17

18 Clarke D, Jones F, Harris R, et al. What outcomes are associated with developing and implementing coproduced interventions in acute healthcare settings? *BMJ Open* 2017; 7:e014650

19 Osborne SP, Radnor Z, Strokosch K. Co-Production and the Co-Creation of Value in Public Services: A suitable case for treatment? *Public Management Review* 2016; 18:5, 639-653

20 Berwick D. Era 3 for Medicine and Health Care. *JAMA* 2016; 315(13):1329-30

21 Batalden M, Batalden P, Margolis P, et al. Coproduction of healthcare service. *BMJ Qual Saf* 2016;25: 509-517

22 Batalden P. Getting more health from healthcare: quality improvement must acknowledge patient coproduction - an essay by Paul Batalden. *BMJ* 2018; 362: k3617

23 Boyle D, Harris M. The challenge of co-production: how equal partnerships between professionals and the public are crucial to improving public services. London: National Endowment for Science, Technology and the Arts 2009

24 SFS. Patientlag [Patient Act]. Stockholm: Sveriges Riksdag 2014: 821

25 Greenhalgh T, Jackson C, Shaw S, et al. Achieving Research Impact Through Co-creation in Community-Based Health Services. *The Milbank Quarterly* 2016; 94(2): 392-429

26 Lindsay C, Pearson S, Batty E, et al. Co-production and social innovation in street-level employability services: Lessons from services with lone parents in Scotland. *International Social Security Review* 2018; 71(4): 33-50

27 Bussu S, Galanti M. Facilitating coproduction: the role of leadership in coproduction initiatives in the UK. *Policy and Society* 2018; 3: 346-367

28 Brown PR, Head BW. Navigating tensions in co-production: a missing link in leadership for public value. *Public Admin* 2018: 1–14

29 Schlappa H, Imani Y. Who is in the lead? New perspectives on leading service co-production. In: Brandsen T, Steen T, Verschuere B (Eds). Co-production and co-creation. Engaging citizens in public services. NY: Routledge 2018

30 Svensson L, Ellström P-E, Brulin G. Introduction: On Interactive Research. *International Journal of Action Research* 2007; 3: 233–249

31 Ellström P-E. Knowledge creation through interactive research: a learning perspective. Paper presented at HHS-07, Jönköping University, Sweden May 8-11 2007

32 Finfgeld-Connett D. Generalizability and transferability of meta-synthesis research findings. *Journal of Advanced Nursing* 2010; 66(2): 246–254

33 Sandelowski M, Barroso J. Handbook for Synthesizing Qualitative Research. New York: Springer Publishing Company 2007

For peer review only - http://bmjopen.bmj.com/site/about/guidelines.xhtml

34 Walsh D, Downe S. Meta-synthesis method for qualitative research: a literature review. *Journal of Advanced Nursing* 2005; 50(2): 204–211
35 Zimmer L. Qualitative meta-synthesis: a question of dialoguing with texts. *Journal of Advanced Nursing* 2006; 53(3): 311–318
36 Best A, Holmes B. Systems thinking, knowledge and action: towards better models and methods. *Evidence & Policy: A Journal of Research, Debate and Practice* 2010; 6(2): 145-159
37 Greenhalgh T, Papoutsi C. Studying complexity in health services research: desperately seeking an overdue paradigm shift. *BMC Medicine* 2018; 16: 95
38 Durose C, Needham C, Mangan C, et al. Generating 'good enough' evidence for co-production. *Evidence & Policy: A Journal of Research, Debate and Practice* 2017; 13(1): 135-151
39 Parrado S, Van Ryzin GG, Bovaird T et al. Correlates of co-production: evidence from a five-nation survey of citizens. *International Public Management Journal* 2013; 16(1): 85-112 doi:10.1080/10967494.2013.796260
40 Uhl-Bien M, Arena M. Leadership for organizational adaptability: A theoretical synthesis and integrative framework. *The Leadership Quarterly* 2018; 29(1): 89-104
41 Drath WH, McCauley CD, Palus CJ et al. Direction, alignment, commitment: toward a more integrative ontology of leadership. *The Leadership Quarterly* 2008; 19(6): 635-653
42 Mohrman S, Gibson CB, Mohrman Jr AM. Doing research that is useful to practice a model and empirical exploration. *Academy of Management Journal* 2001; 44(2):257-375
43 Mulvale G, Moll S, Miatello A, et al. Co-designing health and other public services with vulnerable and disadvantaged populations: Insights from an International Collaboration. *Health Expectations* 2019 https://doi.org/10.1111/hex.12864

Authors' contributions

SK is Principal Investigator and a member of the executive group of the research program. She designed the overall research study, read and contributed to revisions and additions to the manuscript and approved the final version. **KA** is a member of the executive group of the research program. She read and contributed to revisions and additions to the manuscript and approved the final version. **BAG** is a member of the executive group of the research program. She read and contributed to revisions and additions to the manuscript and approved the final version. **ACA** is a member of the executive group of the research program. She read and contributed to revisions and additions to the manuscript and approved the final version. **MO** read and contributed to revisions and additions to the manuscript and approved the final version. **JK** read and contributed to revisions and additions to the manuscript and approved the final version. **JMcG** read and contributed to revisions and additions to the manuscript and approved the final version. **SD** is a member of the executive group of the research program. She read and contributed to revisions and additions to the manuscript and approved the final version. **GR** is a member of the executive group of the research programme. He helped design the overall research study, drafted the original manuscript and contributed to revisions and additions to the manuscript and approved the final version.

Funding statement

This work was supported by Forte: Swedish Research Council for Health, Working Life and Welfare, grant number 2018-01431.

For peer review only - http://bmjopen.bmj.com/site/about/guidelines.xhtml

Competing interests statement

None declared.

Captions

Figure 1 Interactive research

Figure 2 Cross-case analyses: measurement, mechanisms, management and models

For peer review only

For peer review only - http://bmjopen.bmj.com/site/about/guidelines.xhtml

Figure 1 **Interactive research**

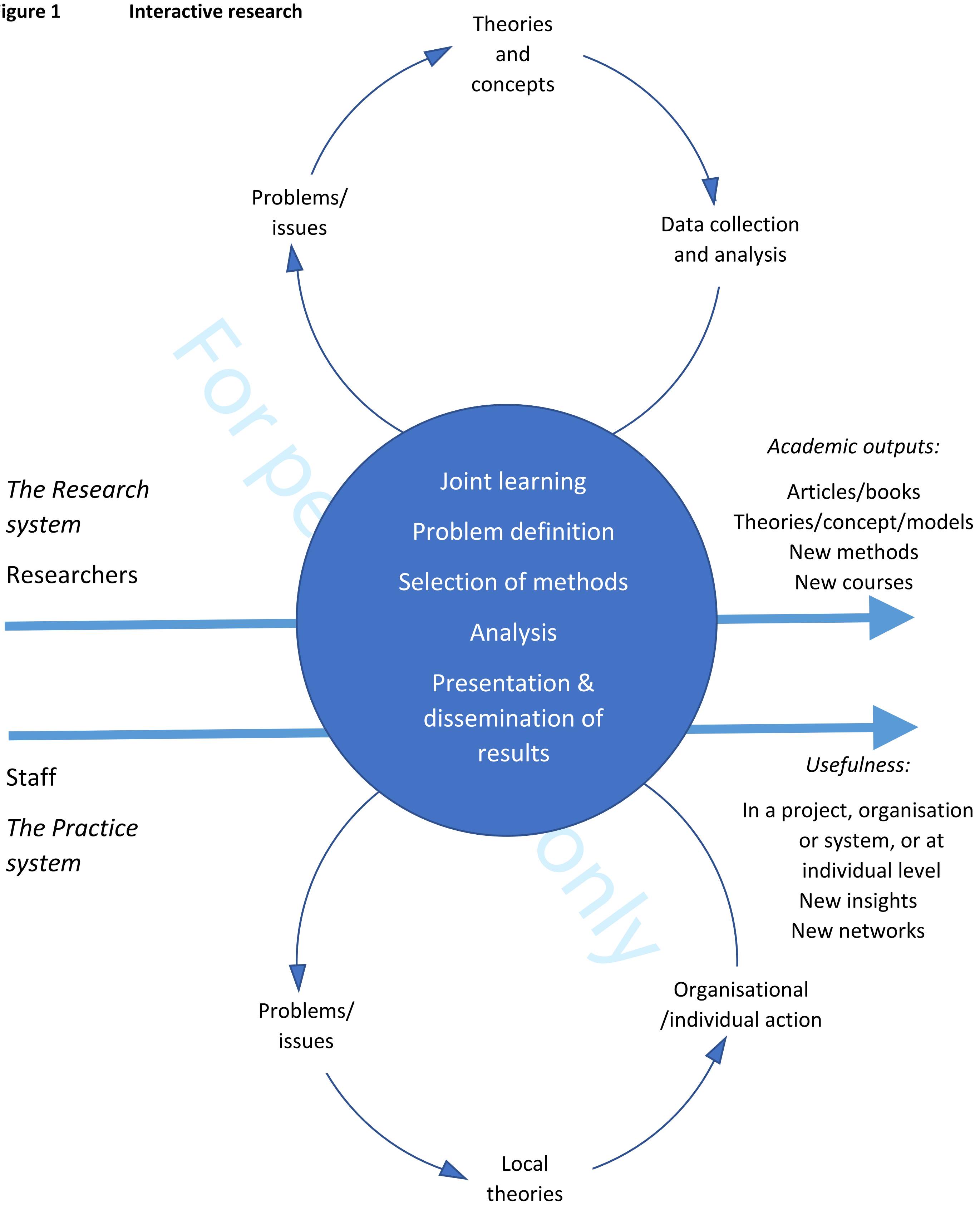

Adapted from Ellström [31] and Svensson et al [30]. Reproduced with permission of Per-Erik Ellström and

Figure 2 **Cross-case analyses: measurement, mechanisms, management and models**

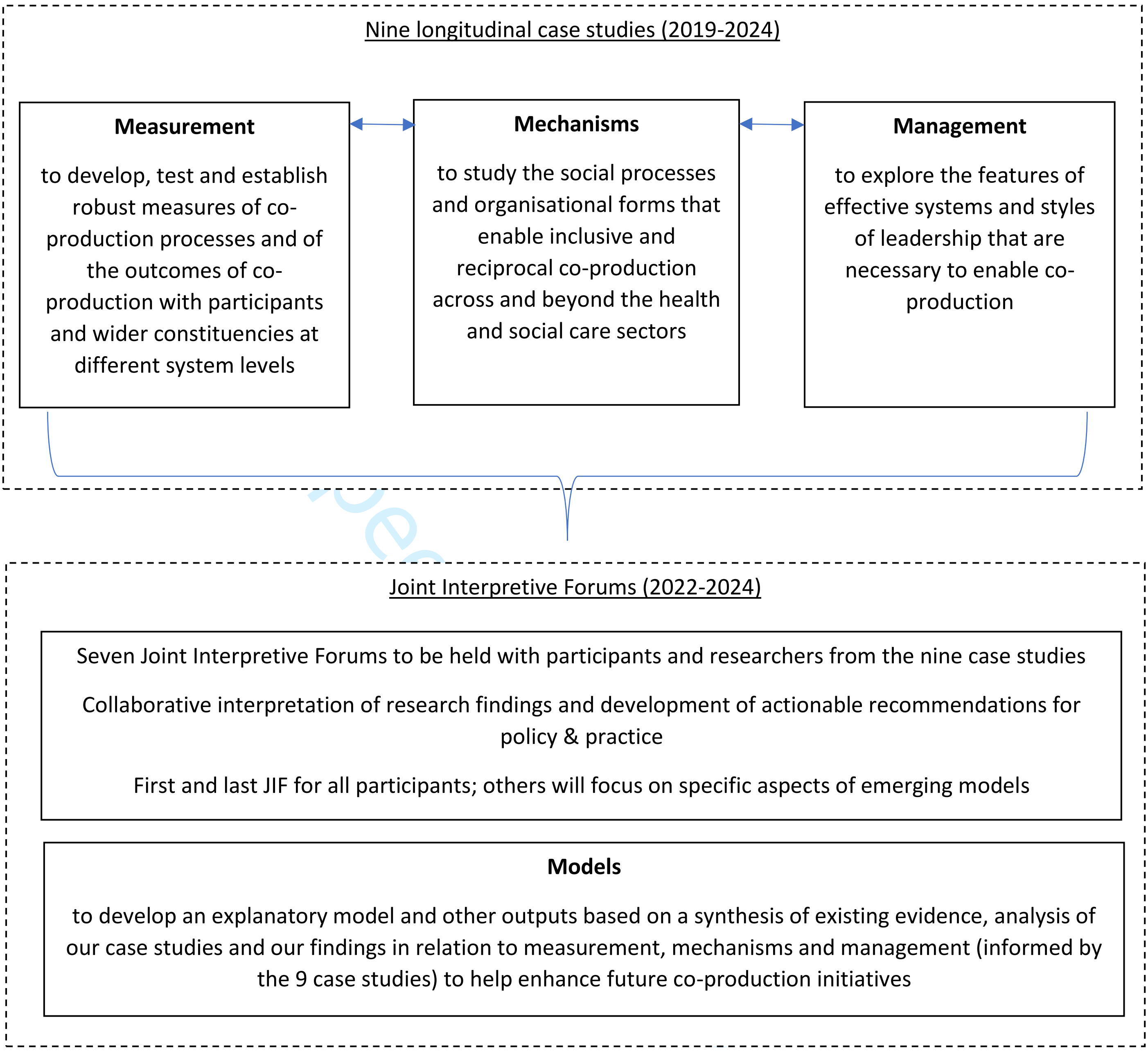

BMJ Open

Exploring, measuring and enhancing the co-production of health and wellbeing at the national, regional and local levels through comparative case studies in Sweden and England: the 'Samskapa' research programme protocol

Journal:	*BMJ Open*
Manuscript ID	bmjopen-2019-029723.R1
Article Type:	Protocol
Date Submitted by the Author:	01-May-2019

Complete List of Authors:	Kjellstrom, Sofia; Jonkoping University, Jonkoping Academy<br>Areskoug-Josefsson, Kristina; Jonkoping University, Jonkoping Academy<br>Gäre, Boel Andersson; Jonkoping University, Jonkoping Academy<br>Andersson, Ann-Christine; Jonkoping University, Jonkoping Academy<br>Ockander, Marlene; Jonkoping University, Jonkoping Academy<br>Kall, Jacob; Djursdala<br>McGrath, Jane; We Coproduce<br>Donetto, Sara; King's College London, Florence Nightingale Faculty of Nursing, Midwifery & Palliative Care<br>Robert, Glenn; King's College London, Florence Nightingale Faculty of Nursing, Midwifery & Palliative Care; Jonkoping University, Jonkoping Academy

**Primary Subject Heading**:	Health services research
Secondary Subject Heading:	Health policy
Keywords:	Health policy < HEALTH SERVICES ADMINISTRATION & MANAGEMENT, Protocols & guidelines < HEALTH SERVICES ADMINISTRATION & MANAGEMENT, co-design, co-production, social care

SCHOLARONE™ Manuscripts

Exploring, measuring and enhancing the co-production of health and wellbeing at the national, regional and local levels through comparative case studies in Sweden and England: the 'Samskapa' research programme protocol

Sofia Kjellström, The Jönköping Academy for Improvement of Health and Welfare, Jönköping University, Sweden*

Kristina Areskoug Josefsson, The Jönköping Academy for Improvement of Health and Welfare, Jönköping University, Jönköping, Sweden

Boel Andersson Gäre, The Jönköping Academy for Improvement of Health and Welfare, Jönköping University, Jönköping, Sweden

Ann-Christine Andersson, The Jönköping Academy for Improvement of Health and Welfare, Jönköping University, Jönköping, Sweden

Marlene Ockander, The Jönköping Academy for Improvement of Health and Welfare, Jönköping University, Jönköping, Sweden

Jacob Käll, Community project leader, Djursdala, Sweden

Jane McGrath, we coproduce, London, United Kingdom

Sara Donetto, Florence Nightingale Faculty of Nursing, Midwifery & Palliative Care, King's College London, London, United Kingdom

Glenn Robert, Florence Nightingale Faculty of Nursing, Midwifery & Palliative Care, King's College London, London, United Kingdom ORCID 0000-0001-8781-6675

Word count (excluding title page, abstract, references, figures and tables): 4,093 words

* Corresponding author: The Jönköping Academy for Improvement of Health and Welfare, Jönköping University, Gjuterigatan 5, Box 1026, 551 11 Jönköping, Sweden, sofia.kjellstrom@ju.se +46 36-10 1313

For peer review only - http://bmjopen.bmj.com/site/about/guidelines.xhtml

Exploring, measuring and enhancing the co-production of health and wellbeing at the national, regional and local levels through comparative case studies in Sweden and England: the 'Samskapa' research programme protocol

Abstract

Introduction: co-creation, co-production and co-design are advocated as effective ways of involving citizens in the design, management, provision and evaluation of health and social care services. Although numerous case studies describe the nature and level of co-production in individual projects, there remain three significant gaps in the evidence base: (a) measures of co-production processes and their outcomes, (b) mechanisms that enable inclusivity and reciprocity, and (c) management systems and styles. By focusing on these issues, we aim to explore, enhance and measure the value of co-production for improving the health and wellbeing of citizens.

Methods and analysis: nine ongoing co-production projects form the core of an interactive research programme ('Samskapa**)** during a six-year period (2019-24). Six of these will take place in Sweden and three will be undertaken in England to enable knowledge exchange and cross-cultural comparison. The programme has a longitudinal case study design using both qualitative and quantitative methods. Cross-case analysis and a sensemaking process will generate relevant lessons both for those participating in the projects and researchers. Based on the findings we will develop explanatory models and other outputs to increase the sustained value (and values) of future co-production initiatives in these sectors.

Ethics and dissemination: all necessary ethical approvals will be obtained from the regional Ethical Board in Sweden and from relevant authorities in England. All data and personal data will be handled in accordance with General Data Protection Regulations. Given the interactive nature of the research programme, knowledge dissemination to participants and stakeholders in the nine projects will be ongoing throughout the six years. External workshops - facilitated in collaboration with participating case studies and citizens - both during and at the end of the programme will provide an additional dissemination mechanism and involve health and social care practitioners, policy makers and third sector organisations.

Keywords: co-production, co-design, healthcare, social care, case study

Strengths and limitations of this study

- moving beyond the study of individual co-production projects and taking a longitudinal, multi-level, cross-case approach to explore the complexities of enabling new forms of relationships to improve health and wellbeing
- a research group from different disciplines and professional backgrounds including interdisciplinary social science, nursing, medical anthropology, medicine, rehabilitation, and sociology
- a close partnership with practitioners and patients/citizens through an interactive research approach will help co-produce and co-evaluate the programme itself
- a potential limitation is the socio-cultural and linguistic differences between the two countries in which the fieldwork will take place

For peer review only - http://bmjopen.bmj.com/site/about/guidelines.xhtml

Introduction

The Swedish health and social care system is struggling - as in many other countries - to balance contemporary challenges of increasing demands and rising costs resulting from demographic changes with the opportunities afforded by technological and scientific advances. Within health system re-design and quality improvement internationally there is currently a 'preoccupation with methods for citizen engagement, public participation and involvement of people with lived experience … Participation has become a distinct cultural and political movement characterised by user involvement in health and social care'.[1] The forms of such involvement span shared decision-making[2], person-centred care and management at the individual patient level through quality improvement[3], research and evaluation at the service and organisational levels[4], and to policy-making at the system level.[5]

The term 'co-production' is increasingly being applied by those working in the health and social care sectors to refer to any of these forms of collaboration between users and providers of services. Nonetheless, principles underpinning the original conceptualisation of co-production - such as mutuality and reciprocity - offer the possibility of fundamentally challenging and changing predominant ways of thinking by moving from focusing solely on the delivery of healthcare and social care and towards co-creating health and wellbeing. This is evidenced in successful, long-standing co-production projects including, for example, the co-creation of a self-hemodialysis service)as undertaken over several years in the Region Jönköping County in Sweden[6], through the work of third-sector organizations like 'We Coproduce' in England[7], and by the development and adaptation internationally of approaches such as Experience-based Co-design (EBCD).[8]

There has recently been renewed academic interest in - and advocacy for - adoption of 'co-production' as a means of co-creating value across the public sector[9,10]. In the healthcare context, co-production is promoted as harnessing the knowledge of patients, carers and staff to make changes about which they care most. It is claimed that 'bringing people together to re-design and improve services as co-producers is re-creating the ways in which public governance, policy and health services are enacted and function'.[1]

Origins and evolution of co-production

Originating in the early 1970s, the term co-production refers to how citizens themselves play an important role in determining the form, delivery and value of public goods and services [11,12]; academic studies of the time were a response to what was seen as a lack of recognition of the role of service users in determining the relative effectiveness of service delivery in different local contexts[13,14]. The creation of time banks - a reciprocity-based work trading system in which hours are the currency where individuals can trade hours of work without paying or being paid for services [15,16] - led to evidence of how collaborative interventions that involve people with long-term psychosocial needs could contribute to improved community links[17].

Academic interest in co-production in different sectors has waxed and waned over the subsequent 50 years but the concept is currently attracting greater interest in public management practices generally[18], including in the healthcare sector specifically. Much of this interest is based on claims of better outcomes and/or efficiency arguments despite empirical evidence remaining limited[19]. With contemporary practices of public sector service delivery highlighting - and espousing the benefits of - efforts to enable co-production has come renewed critical interest from a range of academic disciplines. As summarised by Palmer et al[1], commentators have argued that whilst such interest may reflect a genuine desire to engage citizens in democratic processes, governance and

For peer review only - http://bmjopen.bmj.com/site/about/guidelines.xhtml

decision-making[20], it can also be variously perceived as a means to harness citizen efforts and resources as a replacement for reduced public funding (the 'dark side' of co-production)[21], as a representation of a loss of public value and trust in public sector services and/or a drive to reinvigorate voluntary participation and strengthen community cohesion in response to increasing societal fragmentation.[22] In the healthcare sector specifically, it has been argued that increasing interest in efforts to enable co-production remain uncritical, lacking acknowledgement of the ethical complexities embedded in welfare [and service] relationships.[22] Others have warned against the risk of the term itself losing meaning as it enters mainstream management discourse and practice, thereby losing association with its radical roots.[23] For the purposes of this research programme we are following Osborne et als definition of co-production as 'the voluntary or involuntary involvement of public service users in any of the design, management, delivery and/or evaluation of public services'.[24]

Co-production in the health and social care sector

Notwithstanding definitional issues, Palmer et al argue that we are witnessing 'a political and socio-cultural mindset shift from ''experts know and decide everything'' to ''we need to decide things together''.[1] In the context of health services, people are increasingly characterised as designers, learners, and actors who can take responsibility for their own health and shape the outcomes that they desire from organisations.[8,9,10] An excerpt from a recent manifesto for a third (and moral) era in medicine and health care is typical of this shift: 'Co-production', 'co-design' and person-centred care are among the new watchwords, and professionals and those who train them, should master those ideas and embrace the transfer of control over people's lives to the people."[25] Whilst such contemporary interest in co-production from leaders of quality improvement and Improvement Science in the health and social care context is a relatively recent development[26,27], advocates of the need to study and learn from co-production across the public sector have been doing so for some time.[16,28] A recent Swedish Patient Act supports these trends[29] - as does the latest strategic plan for the National Health Service in England[30] - but there is also a concern that 'co-production' may become a much used but ultimately meaningless term that everyone says that they can and want to do but without understanding its origins or how to practice and evaluate it.

Knowledge gaps

We highlight below three significant and interrelated knowledge gaps pertaining to co-production in the health and social care sectors which our research programme seeks to address: outcomes (measurement), power, power relations and representation (mechanisms), and leadership (management).

Measurement:

Voorberg et al conducted a systematic review of the literature (1987–2013) relating to co-creation/co-production with citizens in public innovation across all sectors. [18] Most studies focused on the identification of influential factors, while hardly any attention was paid to outcomes. Similar conclusions have been drawn by others.[19] Clarke et al systematically reviewed outcomes associated with developing and implementing co-produced interventions in acute healthcare settings; there was a lack of rigorous effectiveness and cost-effectiveness studies at both the service and system levels.[23] Greenhalgh et al report a narrative review of different models of co-creation relevant to community-based health services; they identify key success principles, such as system thinking, processes of co-creation and leadership styles, but note that 'impact is by no means guaranteed'.[31]

For peer review only - http://bmjopen.bmj.com/site/about/guidelines.xhtml

Mechanisms:

Academic reviews of co-production highlight - with some exceptions[32] - little critical engagement with issues of power, power relations, and representation and whether typical patterns of participation serves to make services more or less inclusive (or do they simply reinforce existing social inequalities?).[9,18] In a recent commentary, Batalden et al - while arguing that where healthcare activities are co-produced, services, providers and service users become far more effective agents of change - note that current systems can both support and constrain partnerships between patients and professionals (and that historically this kind of partnership has been unequal).[26]

Management:

Management or leadership of co-production activities should be characterized by the involvement of many stakeholders sharing different perspectives on the same issue. And yet, despite this being described as a founding principle, this is a neglected area as studies of leadership in co-production initiatives are sparse. [33, 34, 35]and. [34,35]. A recent review of coproduction initiatives in the UK suggests four main challenges which require negotiation through different styles of leadership [33]:

- setting priorities of co-production and clarifying goals;
- guaranteeing greater inclusion of vulnerable and disadvantaged populations;
- fostering communication and public accountability; and
- encouraging and supporting innovative practices and cultural changes that move away from traditional challenges of risk aversion.

The review suggested that the leadership of co-production initiatives involves several practices over time, emerges as a complex and collective activity (rather than relying on individual leaders), and likely requires a facilitative leadership system and style.[33]

In summary, despite a recent resurgence of interest in the potential of co-creation and co-production as means not only of maintaining but also improving health and wellbeing in Sweden and England, there remain significant questions regarding how to measure the impacts of such approaches, the mechanisms by which they achieve those impacts and how they can and should be led.

Aim

The overall aim of the Samskapa research programme is to explore, enhance and measure the value of co-production for improving the health and wellbeing of citizens. Our four research objectives are:

1. to develop, test and establish robust measures of co-production processes and of the outcomes of co-production with participants and wider constituencies at different system levels (measurements)
2. to study the social processes and organisational forms that enable inclusive and reciprocal co-production across and beyond the health and social care sectors (mechanisms)
3. to explore the features of effective systems and styles of leadership that are necessary to enable co-production (management)
4. to develop explanatory models and other outputs based on a synthesis of existing evidence and analysis of our empirical findings in relation to 1-3 above in order to help enhance the nine participating projects and future co-production initiatives (model).

For peer review only - http://bmjopen.bmj.com/site/about/guidelines.xhtml

Methods and analysis

This is a six-year, interactive research programme[36] beginning in 2019 that will provide an overarching platform for mixed method evaluations of at least nine case studies of co-production in the health and social care sectors (see table 1 for details of the aims and methods of each of the case studies). The interactive research approach - a form of Participatory Action Research - will place emphasis upon joint learning between the participants and the researchers throughout the entire research process, from the definition of the issues to the analysis and dissemination of findings (see figure 1).[36,37] Cross-case analysis and a sensemaking process with participants - how they interpret and give meaning to their experiences[38] - and researchers from the case studies will lead to practical lessons and outputs to assist practitioners, as well as helping address the key research gaps in the current evidence base as identified above: measurement, mechanisms and management.

As well as responding to recent calls for a 'more multidisciplinary framework, using social-psychological, organizational and institutional theories' to form the basis for future co-production research[9], the programme has a longitudinal design and will use both qualitative (semi-structured interviews, non-participant observation, focus groups) and quantitative methods (for example, patient reported outcome measures and patient reported experience measures within feasibility studies of co-designed interventions) (see table 1). We will seek to establish the impact of co-production, as well as exploring the complexities of delivering and improving health and social care through new forms of relationships and partnerships. Through the interactive research process during the course of the six-year programme, we will be feeding back our emerging findings to the participating case studies as a means of seeking to enhance the impact of their work (figure 1).[39]

[INSERT] Figure 1: Interactive research

Study setting

Fieldwork will be conducted in the context of health and social care provision in Sweden and England. The case studies in Sweden will be undertaken in Region Jönköping County, Kalmas, Stockholm and Gothenburg; the English case studies are in London.

A narrative literature review

We will begin the programme by conducting a systematic scoping review of the literature. Grant and Booth state that a scoping review is a preliminary assessment of the potential size and scope of existing evidence with the aims to identify the nature and extent of research.[40] Our aims will be to explore current knowledge about co-producing health and wellbeing, establish best practice within the health and social care sectors, and inform the Samskapa programme research objectives by reviewing:

- how measures relating to participating in co-production processes and the outcomes of those processes have been developed, by whom and how they have been tested and applied
- the mechanisms (for example, social processes and organisational forms) that enable inclusive and reciprocal co-production
- different individual and collective leadership and management beliefs and practices that enable the co-production of health and wellbeing

For peer review only - http://bmjopen.bmj.com/site/about/guidelines.xhtml

- which and how explanatory models have been developed, tested and applied with the aim of enhancing co-production processes and outcomes.

The databases selected for searching are the Cochrane database of systematic reviews, CINAHL, PsycINFO, Medline, PubMed and Scopus. Our inclusion criteria will be peer reviewed, English-language articles that explicitly relate to co-production or co-design in the health or social welfare context. The findings from the scoping review will directly inform the empirical fieldwork to be undertaken both within and across the nine case studies that together comprise the overall research programme.

The case studies

Nine case studies of co-production will form the basis of our empirical fieldwork (table 1). This set of case studies is an opportunistic sample drawn from co-production projects which members of the research team were either studying - or involved in planning - at the time of our research application; six of the case studies are the subject of planned or ongoing doctoral studies in Sweden or England. There is scope during the six years of the programme to purposively add further case studies as they emerge and in order to help meet our four research objectives. The co-production efforts and processes in each case will be explored by documentary analysis (for example, reviewing key documents such as project protocols and interim reports) as well as qualitative and quantitative methods as relevant to the objectives of each case study (see table 1 for details).

For peer review only

For peer review only - http://bmjopen.bmj.com/site/about/guidelines.xhtml

Table 1 **Initial nine case studies (in Sweden unless stated)**

Case study	Project background	Project objective	Research aim	Research methods
The Esther network	The Esther network - coordinated by Region Jönköping County - recently won the ICT-Enabled Social Innovation (IESI Award) from the EU Science Hub for best initiative supporting active and healthy ageing. The award was for its positive contribution to society as well as its disruptive ICT enabled social innovation potential and high level of service integration. From its origins in 1997 the Esther model has subsequently been adopted and implemented in England, Scotland and Singapore	To improve patient flow and coordination of care	To learn how local co-production approaches can be spread and implemented internationally whilst addressing key issues of fidelity and adaptation	Qualitative study incorporating documentary analysis; interviews with project leaders and participants; and non-participant observation of network events
Patient Compact	At the regional level, an ongoing strategic innovation program in Region Jönköping County (the 'Together' programme) started in 2012 and has developed and expanded over time. The programme is divided into several subprojects, of which - what has become - the national development and implementation of a 'Patient Compact' is one	To transform health care delivery closer to citizens, from hospitals to primary care, from primary care to home care and with a focus on health promotion efforts together with other community actors and citizens themselves	To enhance emerging understandings of co-production as they evolve over time within the Together programme and establish measures relating to co-production and its potential impact on health and wellbeing	Mixed methods evaluation incorporating interviews with patients and staff participants at micro, meso and macro levels; documentary analysis; and participant observation of programme events. Secondary analyses of datasets collected as part of programme (e.g. clinical outcomes, population health)

For peer review only - http://bmjopen.bmj.com/site/about/guidelines.xhtml

We Coproduce (UK)	The origins of We Coproduce as a social enterprise in 2013 are rooted in the recognition, at a mental health hospital in London, that service user involvement was not working, with subsequent development of an independent social enterprise. We Coproduce then also began to work with other community organisations and providers across London to help them embed co-production in their service design and delivery.	Ongoing projects include co-producing a community owned and run radio station; co-producing with frontline mental healthcare staff to support them to make their own films about trauma-based approaches; and co-producing with a local council to embed micro businesses in partnership with bigger local businesses to challenge isolation	To explore challenges and opportunities of service-user led co-production projects in local communities	Qualitative study incorporating documentary analysis; interviews with project leaders and participants; and non-participant observation of co-production events and meetings
Djursdala community project	This case study is funded by an European Union initiative and seeks to identify needs and initiate the development and use of digital solutions that promote the health and wellbeing of a population of approximately 400 citizens in a rural area. Staff at Jönköping Academy are coordinating research into this initiative which is led by the local community	To support rural development projects initiated at the local level in order to revitalize rural areas and enhance local community/rural area	To explore how user-driven digital development can enable co-created and co-produced services that lead to value for a rural area, and whether digital solutions contribute to sustainable development and, if so, in what way	To explore the process of using participatory action research to co-produce methods and solutions with local people from the area, through interviews with community leaders, participants and researchers; and participant observation of community-led events
Chemotherapy-induced peripheral neuropathy (UK)	Some cancer drugs cause damage to nerves, a condition called chemotherapy-induced peripheral neuropathy (CIPN). The most common symptoms,	To co-design and test an intervention to reduce falls and injuries and improve functional status and	To study how co-designed interventions can be developed and put in place early to prevent subsequent CIPN-related falls and injuries,	Feasibility of randomised controlled trial (RCT) with embedded process evaluation (will include semi-structured telephone interviews with all

For peer review only - http://bmjopen.bmj.com/site/about/guidelines.xhtml

	felt mainly on hands and feet, are numbness, tingling, pain, muscle weakness, and/or sensitivity to cold. People with CIPN can have functional difficulties in carrying out tasks involving their hands and feet. It is important to prepare patients about the possibility of developing CIPN to help them recognise and report symptoms early so healthcare professionals (HCPs) can support them	quality of life among individuals with CIPN	reduce costs to healthcare systems and lessen the burden on HCPs and services	patient participants (n=40) to assess acceptability of the intervention and evaluation methods). Patients will complete outcome measures (early symptom reporting; reduction in symptoms and self-efficacy in managing symptoms; improved functional status; quality of life) at various timepoints
Learning café: cardiac care	A Learning Café project is underway where people with cardiac care needs come together to collectively discuss how they can improve different aspects of their health and wellbeing. Clinical measures - as well as patient-reported outcome measures and patient-reported experience measures - are being co-designed with patients and families and professionals. In addition a co-designed conceptual model of the Learning Café which can be adapted to other groups of patients with chronic disease is being developed	To explore whether, how and why the co-production of healthcare services, particularly for individuals (and their families) with cardiac care needs, can contribute to high quality care	To explore what role motivation plays for patients, family members and healthcare professionals when co-producing healthcare	Mixed methods study incorporating patient surveys (sense of security in everyday life, patient satisfaction); focus groups and semi-structured interviews with patients and professionals; patient diaries; and non-participant observation

For peer review only - http://bmjopen.bmj.com/site/about/guidelines.xhtml

Disabled children & adolescents	This study is mainly taking place in a not-profit organization in Solberga By, near Stockholm, and is drawing upon an action research design to study local quality improvement initiatives to enhance individual support to children with intellectual disability living in special care residence. This includes studying if and how the children's role as co-producers is reinforced by these initiatives	To enhance staff capacity to design, test and follow up individual support to children with intellectual disabilities	The overall aim is to explore the usefulness of integrating improvement knowledge and the International Classification of Functioning, Disability and Health in staff working procedures to improve goal fulfilment and coproduction for children living in special care residence	Realistic evaluation study design including data collection from observations, behavior and function assessments, field notes from staff sessions, QI-documentation and focus groups
Therapeutic engagement on an acute psychiatric ward (UK)	Therapeutic engagement has long been regarded as the essence of mental health nursing. Its benefits are well documented: inpatients who are socially engaged adjust better to community life, have greater symptom improvements during treatment and exhibit fewer violent and aggressive behaviours. Nurses who spend more therapeutic time with patients have greater job satisfaction and take fewer sick days, which may reduce the costly use of unfamiliar agency nurses. Despite this, research spanning 35 years shows that	To empower a service-user group to take a lead role, and in partnership with NHS staff, co-design and implement an intervention to improve nurse-patient therapeutic engagement on acute mental health wards	To assess the project in terms of improvements in the amount, type and quality of nurse-patient engagement; improved service user/service provider relations; and the fostering of a culture of collaborative working/research practices within a psychiatric ward	Mixed methods evaluation incorporating interviews with patients and staff participants; non-participant observation of co-design events; event questionnaires; and a pre-post test design on an intervention and control ward using structured qualitative and quantitative observations, a self-report measure and data from ward registers to assess type, quality and amount of engagement

For peer review only - http://bmjopen.bmj.com/site/about/guidelines.xhtml

	just 4-12% of nurses' time was spent on therapeutic activities			
Learning health system for severe mental illness	In the department for psychosis at Sahlgrenska University Hospital in Gothenburg a Learning Health System has begun to be developed and tested along with patients, case-managers and the management team. Patients and families are active participants in considering system design, user-experience-design, choice of outcome measures and development of care processes	To enable learning throughout the whole "system" and continuous improvement	To explore the role of patients in, firstly, the development of data-visualization-design and how this impacts upon learning both for the patient and their case manager, and secondly in evaluating outcome measures useful for the patient and their case-manager in ongoing treatment	Mixed methods study incorporating surveys; interviews; and non-participant observation

For peer review only

In the following sections we outline how our scoping review and empirical fieldwork in the nine case studies will combine to inform our four research objectives.

Measurement

The scoping review (see above) will explore how measures relating to the different forms of value of participating in co-production processes and the outcomes of those processes have been developed, by whom and how they have been tested and applied (for example, Durose et al[41]). We will explore the usefulness of existing measures in the case studies by observing their use (if any) in practice and facilitating interactive workshops with case study leaders on this topic (particularly on how measures and co-evaluation of processes and outcomes are integrated in the cases). The precise nature of the observational fieldwork to be undertaken will vary by case study. In most of the cases this will be through non-participant observation of key meetings and events (and will incorporate any discussions relating to measures). In a minority of cases more structured forms of observation will be conducted and/or participant observation will be used. We will then co-design with stakeholders and users of the relevant services new generic measures of co-production (through an interactive process partly informed by the scoping review findings) and then test them over time in the case studies to assess their pragmatic usefulness and generalizability.

How - and to what extent - client/patient involvement is present and acted upon in the design and evaluation of outcome measures, as well as the sustainability and understanding of the value of the outcomes, will also be qualitatively explored in the case studies. We will consider the gender and sociodemographic differences (for example, age, gender) amongst those who actively choose to participate in the co-production case studies as this may influence both the chosen measurements and the outcomes.[42] The programme will provide an increased understanding of how to develop and use measures within – as well as to evaluate the outcomes of - co-production projects by illuminating how specific processes in co-production relate to measured outcomes (see 'mechanisms' below).[42]

Mechanisms

We will study the concepts and practices of co-production to identify mechanisms (for example, social processes and organisational forms) that contribute to or hinder the development of values and actions that enable inclusive and reciprocal co-production. Firstly, our scoping review (see above) will identify previous studies (for example, Palmer et al[1]) which have sought to explore such mechanisms. The review will establish what is already known about key issues such as power and levels of representativeness, and any interventions or modifications that have attempted to resolve these. Secondly, informed by the narrative review findings, we will then draw on our emerging empirical findings relating to the local conceptualisations and practices in the nine case studies to design complementary or alternative ways of working. We will subsequently observe the implementation of these in one or more of the case studies to assess whether and how they enhance the co-production efforts.

Management/leadership

Our scoping review will establish what is known about different individual and collective leadership and management assumptions that enable the co-production of health and wellbeing. Leadership will be framed as complex, interactive and dynamic, and analysed in a way that contributes to generative learning and theoretical transferability.[43,44] Studying leadership needs to shift away

For peer review only - http://bmjopen.bmj.com/site/about/guidelines.xhtml

from a focus on leaders and followers styles and towards a system of processes through which a collective endeavour unfolds. The sensemaking and assumptions about leaders and leadership has a central role in how leadership is enacted and can be evaluated, developed and influenced.[45] This approach builds on a belief that individual and collective assumptions about leadership shape how team organizational members work with leadership within an organization. Our longitudinal study design will enable us to address whether leadership assumptions vary across cases, how they change and develop over time and how people in different positions of power participate in various leadership activities and sense making processes?[45,46] We will explore leadership across the nine case studies through a range of methods including semi-structured interviews, surveys, and non-participant observation of meetings and events. Workshops will also be facilitated with researchers and key actors to elicit their views on how leadership has been enacted in each of the cases. The results will be summarized in a dynamic system assessment tool that seeks to aid understanding of how to analyse and improve co-production leadership.

Models

Based on the multi-level and longitudinal case studies in different health and social care settings in Sweden and England we will conduct a meta-synthesis.[46,47,48,49,50] We will use theoretical perspectives - identified and selected through our scoping review (see above) - and systems thinking to make sense of the context and cases.[43,51] In the meta-synthesis we will explore patterns in both the qualitative and quantitative data from across the different case studies and examine and compare differences and similarities across these. To inform this cross-case analysis - in addition to the 'within' case fieldwork outlined in table 1 - longitudinal semi-structured interviews will be conducted at least annually during the six years of the programme with key leaders of each of the nine case studies. These will focus on our four objectives - measurement, mechanisms, management and models - and how practices of co-production are being enacted over time. We will synthesise the findings from the 'within' and 'across' case study analyses to identify relevant themes, similarities, and differences between the cases (figure 2).

[INSERT] [Figure 2: Cross-case analyses]

This work will develop explanatory models for successful co-production through analysis of all cases and synthesis of our findings in relation to measurement, mechanisms and management. Further outputs will include the co-design of tools to enable those participating in co-production projects to reflect upon the maturity of their efforts and how their work might be enhanced. The narrative review will establish which and how explanatory models have been developed, tested and applied with the aim of enhancing co-production processes and outcomes. As a part of our interactive approach we will convene and facilitate multiple stakeholders meetings (with leaders, participants and researchers of the 9 case study projects) in the format of Joint Interpretive Forums (JIFs) - a form of group discussion which aims to foster 'perspective taking' and joint decision making - to enable the collaborative interpretation of both our review and empirical research findings and the development of actionable recommendations for policy and practice.[52] The first JIF will be convened at the end of year three; a JIF will then be held at 6-month intervals for the remaining duration of the programme (7 JIFs in total). The initial and final JIF will be open to all participants whilst the others will focus on specific aspects of the emerging model(s) and invitees will be selected as appropriate. Informed by the findings from above all the JIFs will be held in Sweden. Importantly,

For peer review only - http://bmjopen.bmj.com/site/about/guidelines.xhtml

all PhD students who are studying one of the case studies will be integral members of this ongoing sensemaking process giving them further personal development opportunities.

Patient and Public Involvement

Jönköping Academy for Improvement of Health & Welfare (JA) is the host of the research programme and has an established tradition of interactive research where knowledge is created in the interaction between theory and practice. 'Practice' has traditionally been represented by professionals in this model but in this programme we will use and expand this to include citizens and patients as full partners (see Figure 1). Processes to enhance partnership working through ongoing, joint design of the specific research materials and methods to be applied in each of the case studies will therefore be both a result and a phenomenon for study in the programme. As part of our ongoing interactive approach, members of the research team have identified several 'charters for co-production' (tools to aid reflective practice within co-production projects) from the UK and the US and had these translated into Swedish. These will form the basis for reflective dialogues which will test the appropriateness of applying these various materials in the Swedish context.

Through conversations with public and third-sector organizations already engaged in co-production in health and social care, the Region Jönköping County in 2016 decided to financially support development of an International Centre for Co-production hosted by JA as a sister Centre to a similar Centre at the Dartmouth Institute, New Hampshire in the United States. In 2017 Jönköping University made a strategic decision to support the research capacity in the Centre at JA through investing in senior research positions in co-production and a project leader. In addition, trade unions and small and medium size enterprises have been involved in ongoing discussions. Processes to integrate user and public representatives are in progress and they can contribute in several ways; in the co-design of service innovations but also in interactive research processes to assure the relevance of questions and the validity of results. The Centre provides infrastructure that supports the involvement of patients and citizens in research processes as well as in practice. From the UK, a community-based organisation 'We Coproduce' will form one of our case studies and leaders of this organisation will also engage with the doctoral and post-doctoral students in a regular series of practice-based workshops. The leaders of the Djursdala and 'We Coproduce' case studies have contributed to and are co-authors of this protocol.

Partnership working is an inherent feature of each of the nine case studies of co-production which we will initially be studying. At the programme level, a Partnership Learning Group will consist of all the current authors, participating post-doctoral researchers and doctoral students, as well as practitioners and citizens from the six Swedish case studies. This group will meet throughout the six-year programme. Through dialogue, cross-case study sharing of knowledge and development of further research questions and thoughts of interest, the members will help co-design, co-produce, and co-evaluate the program as a whole; these interactions will be supported by specific seminars. The Group will design dissemination workshops within each of their case studies to enhance cross-case study knowledge creation and networking.

Ethics and dissemination:

Ethics

Ethical approval will be obtained from the regional Ethical Board in Sweden for all of the case studies where this is applicable and from the relevant authorities in England for the case studies there. All

For peer review only - http://bmjopen.bmj.com/site/about/guidelines.xhtml

data and personal data will be handled in accordance with the new European General Data Protection Regulation (GDPR) (EU) 2016/679). Given the need to respect the integrity, autonomy, and privacy of the participants, it is important to acknowledge that ethical considerations and principles may exert an influence on the research design. Informed consent will be accomplished by mutual communication where the researcher provides accurate information and listens to the individual participants in order to make sure that they comprehend and make voluntary choices to participate, not only at initial recruitment but also throughout participation in the project. This is particularly important for vulnerable groups,[53] some of which will be participating in this programme (see final three projects in table 1). Whilst the interactive research design (i.e. including patients, staff and stakeholders in the whole research process) can be challenging to Ethical Boards - posing difficulties in detailing the nature and timing of the research to be undertaken beforehand - the research team have extensive experience of successfully navigating these ethical processes in both Sweden and England.

Dissemination

Our intention is to engage strategically with five audiences with whom we will deepen and sustain existing - and create new - relationships to help inform our ongoing research and to provide opportunities to create positive change in health and social care services:

- leaders in the international, national and regional planning of health and social care services
- educators developing the next generation of health and social care professionals
- professional bodies and trade unions
- citizens as they access, use and shape services
- research funders

We will adopt a structured approach to mapping key organisations, networks and opinion leaders at international, national and regional levels; this will be part of our work both in terms of developing a communications strategy in year one of the programme and to help us identify diverse and influential members of our Advisory Board. As part of this approach we will engage with national and regional leaders of health and social care services through contact with the 'Swedish Association of Local Authorities and Regions' (SALAR). The Department for Health & Social Care at SALAR has responsibility, for example, for supporting the development of elderly care, social care, public health, disability, quality and safety, and equity. SALAR also coordinates several national networks of leaders from across all Swedish regions and counties providing opportunities to share our findings and outputs to contribute to policy discussions and practice.

A further prime opportunity for dissemination is through involving educators and students - often mid-career professionals - involved with a Master programme in 'Leadership for Improvement of Health and Welfare' developed and run at the Jönköping Academy, Jönköping University; this will enable us to engage with students involved in leading improvement of care throughout Sweden. We also have connections with national bodies with an interest in integrating knowledge on co-production into different levels of education. A process of interaction with professional bodies and trade unions started in March 2018 and will enable further collaboration during the research programme. Citizens are both key participants in - and an important audience for - the programme and will be invited to participate and integrated throughout our work; we will also approach Swedish patient organizations. Whilst there is no national umbrella patient organization, there are many disease/condition specific patient advocacy groups which offer opportunities for testing and scaling up co-production initiatives. Finally, contact has also been initiated by the funder with those leading

For peer review only - http://bmjopen.bmj.com/site/about/guidelines.xhtml

parallel research programs on co-production taking place in Sweden raising the potential for increasing adoption of the findings within and across programmes.

In combination with the interactive research approach outlined above, the proposed involvement of researchers, practitioners and citizens will enable fruitful ways of dissemination and impact throughout the six year Samskapa programme, enhancing the likelihood that co-production can be a catalyst for new forms of relationships to deliver and improve health and wellbeing.

References

1 Palmer V, Weavell W, Callander R, et al. The participatory Zeitgeist - identification of mechanisms of change and the development of an explanatory theoretical model for a new era of healthcare quality improvement. *Medical Humanities* Published Online First: 28 June 2018. doi: 10.1136/medhum-2017-011398

2 Barry MJ, Edgman-Levitan S. Shared decision making - the pinnacle of patient-centered care. *New England Journal of Medicine* 2012 Mar 1;366(9):780-1

3 Entwistle VA, Watt IS. Treating patients as persons: a capabilities approach to support delivery of person-centered care. *The American Journal of Bioethics* 2013 Aug 1;13(8):29-39

4 Brett J, Staniszewska S, Mockford C, et al. Mapping the impact of patient and public involvement on health and social care research: a systematic review. *Health Expectations* 2014 Oct;17(5):637-50.

5 Oxman AD, Lewin S, Lavis JN, Fretheim A. SUPPORT Tools for evidence-informed health Policymaking (STP) 15: Engaging the public in evidence-informed policymaking. *Health Research Policy and Systems* 2009 Dec;7(1):S15

6 Shinkman R. Is 'empowered dialysis' the key to better outcomes? *NEJM Catalyst* 2018 (15 March)

7 we coproduce. https://www.wecoproduce.com/, accessed 23/01/19

8 Robert G, Cornwell J, Locock L, et al. Patients and staff as co-designers of health care services. *British Medical Journal* 2015; 350:g7714

9 Brandsen T, Steen T, Verschuere B (Eds). Co-production and co-creation. Engaging citizens in public services. NY: Routledge 2018

10 Pestoff V, Brandsen T, Verschuere B (Eds). New Public Governance, the Third Sector and Co-Production. NY, Routledge 2012

11 Ostrom E, Parks RB, Whitaker GB, et al. The public service production process: a framework for analysing police services. *Policy Studies Journal* 1978; 7: 381-9

12 Alford J. The Multiple Facets of Co-Production: Building on the work of Elinor Ostrom. *Public Management Review* 2014; 16(3):299-316

13 Realpe A, Wallace LM. What is co-production? London: The Health Foundation 2016

14 Alford J. Engaging public sector clients, from service delivery to co-production. NY: Palgrave 2009

15 Boyle D, Clark S, Burns S. Hidden work: co-production by people outside paid employment London: Joseph Rowntree Foundation 2006

16 Needham C, Carr S. Co-production: an emerging evidence base for adult social care transformation. London: Social Care Institute for Excellence 2009

17 Alford J, Freijer L. Public management and co-production. In: Brandsen T, Steen T, Verschuere B (Eds). Co-production and co-creation. Engaging citizens in public services. NY: Routledge 2018

For peer review only - http://bmjopen.bmj.com/site/about/guidelines.xhtml

18 Voorberg WH, Bekkers VJJM, Tummers LG. A Systematic Review of Co-Creation and Co-Production: Embarking on the social innovation journey. *Public Management Review* 2015; 17:9: 1333-1357

19 Loeffler E, Bovaird T. User and Community Co-Production of Public Services: What Does the Evidence Tell Us? *International Journal of Public Administration* 2016; 39(13): 1006-1019

20 Brandsen T, Honingh M. Distinguishing Different Types of Co-production: A Conceptual Analysis Based on the Classical Definitions. *Public Administration Review* 2016; 76(3):427-35

21 Fotaki M. Co-production under the Financial Crisis and Austerity: a means of democratizing Public Services or a race to the bottom? *Journal of Management Inquiry* 2015; 24(4):433-8

22 Cribb A, Gewirtz S. New welfare ethics and the remaking of moral identities in an era of user involvement. *Globalisation Societies and Education* 2012; 10(4):507-17

23 Clarke D, Jones F, Harris R, et al. What outcomes are associated with developing and implementing coproduced interventions in acute healthcare settings? *BMJ Open* 2017; 7:e014650

24 Osborne SP, Radnor Z, Strokosch K. Co-Production and the Co-Creation of Value in Public Services: A suitable case for treatment? *Public Management Review* 2016; 18:5, 639-653

25 Berwick D. Era 3 for Medicine and Health Care. *JAMA* 2016; 315(13):1329-30

26 Batalden M, Batalden P, Margolis P, et al. Coproduction of healthcare service. *BMJ Qual Saf* 2016;25: 509-517

27 Batalden P. Getting more health from healthcare: quality improvement must acknowledge patient coproduction - an essay by Paul Batalden. *BMJ* 2018; 362: k3617

28 Boyle D, Harris M. The challenge of co-production: how equal partnerships between professionals and the public are crucial to improving public services. London: National Endowment for Science, Technology and the Arts 2009

29 SFS. Patientlag [Patient Act]. Stockholm: Sveriges Riksdag 2014: 821

30 NHS England. The NHS Long Term Plan. 2019

31 Greenhalgh T, Jackson C, Shaw S, et al. Achieving Research Impact Through Co-creation in Community-Based Health Services. *The Milbank Quarterly* 2016; 94(2): 392-429

32 Lindsay C, Pearson S, Batty E, et al. Co-production and social innovation in street-level employability services: Lessons from services with lone parents in Scotland. *International Social Security Review* 2018; 71(4): 33-50

33 Bussu S, Galanti M. Facilitating coproduction: the role of leadership in coproduction initiatives in the UK. *Policy and Society* 2018; 3: 346-367

34 Brown PR, Head BW. Navigating tensions in co-production: a missing link in leadership for public value. *Public Admin* 2018: 1–14

35 Schlappa H, Imani Y. Who is in the lead? New perspectives on leading service co-production. In: Brandsen T, Steen T, Verschuere B (Eds). Co-production and co-creation. Engaging citizens in public services. NY: Routledge 2018

36 Svensson L, Ellström P-E, Brulin G. Introduction: On Interactive Research. *International Journal of Action Research* 2007; 3: 233–249

37 Aagaard Nielsen K, Svensson L. (eds) Action and Interactive Research. Beyond practice and theory. 2006 Maastricht: Shaker Publishing

38 Weick K, Sutcliffe KM, Obstfeld D. Organizing and the process of sensemaking. *Organization Science* 2005; 16(4): 409–421

39 Ellström P-E. Knowledge creation through interactive research: a learning perspective. Paper presented at HHS-07, Jönköping University, Sweden May 8-11 2007

40 Grant MJ, Booth A. A typology of reviews: an analysis of 14 review types and associated methodologies. *Health Information & Libraries Journal* 2009 Jun;26(2):91-108

For peer review only - http://bmjopen.bmj.com/site/about/guidelines.xhtml

41 Durose C, Needham C, Mangan C, et al. Generating 'good enough' evidence for co-production. *Evidence & Policy: A Journal of Research, Debate and Practice* 2017; 13(1): 135-151

42 Parrado S, Van Ryzin GG, Bovaird T et al. Correlates of co-production: evidence from a five-nation survey of citizens. *International Public Management Journal* 2013; 16(1): 85-112 doi:10.1080/10967494.2013.796260

43 Greenhalgh T, Papoutsi C. Studying complexity in health services research: desperately seeking an overdue paradigm shift. *BMC Medicine* 2018; 16: 95

44 Uhl-Bien M, Arena M. Leadership for organizational adaptability: A theoretical synthesis and integrative framework. *The Leadership Quarterly* 2018; 29(1): 89-104

45 Probert J, Turnbull James K. Leadership development: crisis, opportunities and the leadership concept. *Leadership* 2011; 7: 137–150

46 Drath WH, McCauley CD, Palus CJ et al. Direction, alignment, commitment: toward a more integrative ontology of leadership. *The Leadership Quarterly* 2008; 19(6): 635-653

47 Finfgeld-Connett D. Generalizability and transferability of meta-synthesis research findings. *Journal of Advanced Nursing* 2010; 66(2): 246–254

48 Sandelowski M, Barroso J. Handbook for Synthesizing Qualitative Research. New York: Springer Publishing Company 2007

49 Walsh D, Downe S. Meta-synthesis method for qualitative research: a literature review. *Journal of Advanced Nursing* 2005; 50(2): 204–211

50 Zimmer L. Qualitative meta-synthesis: a question of dialoguing with texts. *Journal of Advanced Nursing* 2006; 53(3): 311–318

51 Best A, Holmes B. Systems thinking, knowledge and action: towards better models and methods. *Evidence & Policy: A Journal of Research, Debate and Practice* 2010; 6(2): 145-159

52 Mohrman S, Gibson CB, Mohrman Jr AM. Doing research that is useful to practice a model and empirical exploration. *Academy of Management Journal* 2001; 44(2):257-375

53 Mulvale G, Moll S, Miatello A, et al. Co-designing health and other public services with vulnerable and disadvantaged populations: Insights from an International Collaboration. *Health Expectations* 2019 https://doi.org/10.1111/hex.12864

For peer review only

Acknowledgements

We are grateful to the following doctoral students for participating in the Samskapa research programme and providing details of their studies as presented in table 1: Andreas Gremyr (Jönköping University), Sarah McAllister (King's College London), Sofia Persson (Jönköping University), Anne-Marie Suutari (Jönköping University), Mary Tanay (King's College London) and Pontus Wallin (Jönköping University).

Authors' contributions

SK is Principal Investigator and a member of the executive group of the research program. She designed the overall research study, read and contributed to revisions and additions to the manuscript and approved the final version. **KAJ** is a member of the executive group of the research program. She read and contributed to revisions and additions to the manuscript and approved the final version. **BAG** is a member of the executive group of the research program. She read and contributed to revisions and additions to the manuscript and approved the final version. **ACA** read

For peer review only - http://bmjopen.bmj.com/site/about/guidelines.xhtml

and contributed to revisions and additions to the manuscript and approved the final version. **MO** read and contributed to revisions and additions to the manuscript and approved the final version. **JK** read and contributed to revisions and additions to the manuscript and approved the final version. **JMcG** read and contributed to revisions and additions to the manuscript and approved the final version. **SD** is a member of the executive group of the research program. She read and contributed to revisions and additions to the manuscript and approved the final version. **GR** is a member of the executive group of the research programme. He helped design the overall research study, drafted the original manuscript and contributed to revisions and additions to the manuscript and approved the final version.

Funding statement

This work is supported by Forte: Swedish Research Council for Health, Working Life and Welfare, grant number 2018-01431.

Competing interests statement

None declared.

Captions

Figure 1 Interactive research

Figure 2 Cross-case analyses: measurement, mechanisms, management and models

For peer review only

Figure 1 **Interactive research**

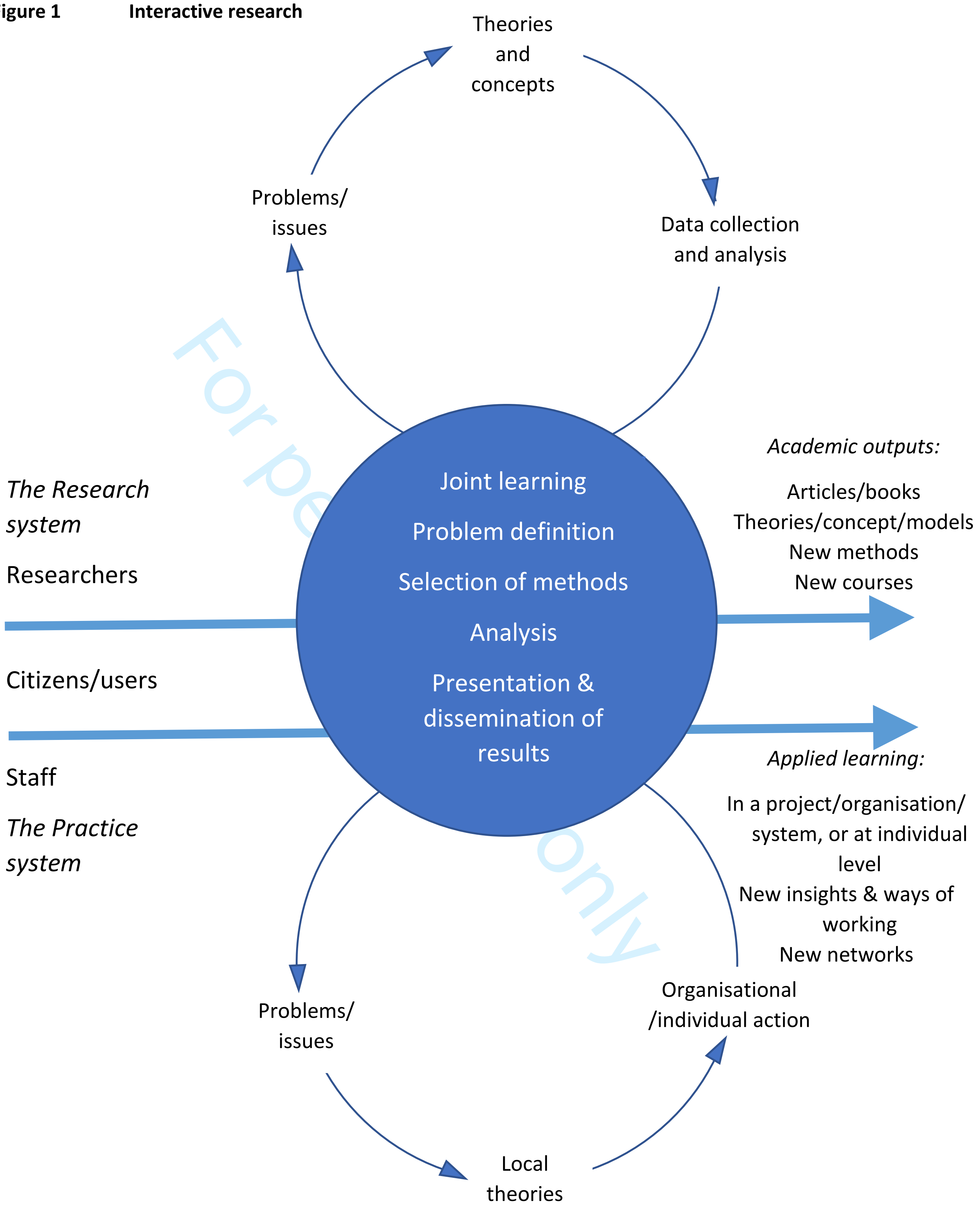

Adapted from Ellström [39] and Svensson et al [36]. Reproduced with permission of Per-Erik Ellström and Budrich UniPress.

For peer review only

Figure 2 Cross-case analyses: measurement, mechanisms, management and models

'Within' and 'across' case analysis of nine longitudinal case studies (2019-2024)

Measurement

to develop, test and establish robust measures of co-production processes and of the outcomes of co-production with participants and wider constituencies at different system levels

Mechanisms

to study the social processes and organisational forms that enable inclusive and reciprocal co-production across and beyond the health and social care sectors

Management

to explore the features of effective systems and styles of leadership that are necessary to enable co-production

Joint Interpretive Forums (2022-2024)

Seven Joint Interpretive Forums to be held with participants and researchers from the nine case studies

Collaborative interpretation of research findings and development of actionable recommendations for policy & practice

First and last JIF for all participants; others will focus on specific aspects of emerging models

Models

to develop an explanatory model and other outputs based on a synthesis of existing evidence, analysis of our case studies and our findings in relation to measurement, mechanisms and management (informed by the 9 case studies) to help enhance future co-production initiatives
